# Novel Picornavirus in Turkey Poults with Hepatitis, California, USA

**DOI:** 10.3201/eid1703.101410

**Published:** 2011-03

**Authors:** Kirsi S. Honkavuori, H. L. Shivaprasad, Thomas Briese, Craig Street, David L. Hirschberg, Stephen K. Hutchison, W. Ian Lipkin

**Affiliations:** Author affiliations: Columbia University, New York, New York, USA (K.S. Honkavuori, T. Briese, C. Street, D.L. Hirschberg, W.I. Lipkin);; California Animal Health and Food Safety Laboratory System, Tulare, California, USA (H.L. Shivaprasad);; 454 Life Sciences, Branford, Connecticut, USA (S.K. Hutchison)

**Keywords:** turkeys, avian, hepatitis, picornavirus, immunohistochemistry, viruses, United States, California, expedited, research

## Abstract

To identify a candidate etiologic agent for turkey viral hepatitis, we analyzed samples from diseased turkey poults from 8 commercial flocks in California, USA, that were collected during 2008–2010. High-throughput pyrosequencing of RNA from livers of poults with turkey viral hepatitis (TVH) revealed picornavirus sequences. Subsequent cloning of the ≈9-kb genome showed an organization similar to that of picornaviruses with conservation of motifs within the P1, P2, and P3 genome regions, but also unique features, including a 1.2-kb sequence of unknown function at the junction of P1 and P2 regions. Real-time PCR confirmed viral RNA in liver, bile, intestine, serum, and cloacal swab specimens from diseased poults. Analysis of liver by in situ hybridization with viral probes and immunohistochemical testing of serum demonstrated viral nucleic acid and protein in livers of diseased poults. Molecular, anatomic, and immunologic evidence suggests that TVH is caused by a novel picornavirus, tentatively named turkey hepatitis virus.

Turkey viral hepatitis (TVH) is a highly infectious disease affecting young turkey poults. The disease is often subclinical, causing minor histologic lesions, and becomes overt when the animals are stressed, resulting in varying rates of illness and death (*1*). Mortality rates of up to 25% have been reported (*2*). Diagnosis is based on characteristic lesions in the liver, which include multifocal necrosis and mononuclear inflammatory cell infiltrates (*3**,**4*). Similar lesions may be found in the pancreas. Clinical signs include anorexia, depression, diarrhea, and weight loss compatible with a diagnosis of enteritis, the second most common diagnosis made in turkey poults throughout the United States. Although we cannot with confidence estimate the specific burden of TVH, its economic effects are likely substantial; in the United States, turkey production was valued at $3.71 billion in 2007. The identification of a pathogen and development of specific diagnostics will lead to better understanding of the economic consequences and other effects of TVH.

The disease has been experimentally reproduced in turkey poults by inoculation with material derived from affected animals (*1**–**4*). A viral basis for TVH has been presumed since its initial description in 1959 because the causative agent passed through 100-nm membranes, was acid stable, was not affected by antimicrobial drugs, and could be propagated in the yolk sac of embryonated chicken eggs (*3**–**5*). Icosahedral particles of 24 to 30 nm have been found by electron microscopy (EM) in liver lesions of birds (*6*) as well as in embryonated turkey eggs (*1*) that have been inoculated with material derived from affected birds; however, no agent has been consistently implicated (*7**,**8*).

## Materials and Methods

### Animals

Samples from healthy and diseased turkey poults were collected from February 2008 through January 2010 from 8 commercial flocks in California, USA. Flock sizes ranged from 22,500 to 40,000 birds. Clinical signs in diseased poults included anorexia, lethargy, diarrhea, and increased mortality rates. Postmortem analyses revealed livers with white foci and, occasionally, pale patchy areas in the pancreas. Histopathologic examination showed necrosis of hepatocytes and acinar cells of the pancreas and inflammation.

### High-throughput Pyrosequencing

Total RNA was extracted from 4 sick 25-day-old turkey poults by using TRI Reagent (Molecular Research Center, Inc. Cincinnati, OH, USA) and treated with DNaseI (Ambion, Austin, TX, USA). Two micrograms of DNaseI-treated RNA was reverse transcribed by using Superscript II (Invitrogen, Carlsbad, CA, USA) and random octamer primers with an arbitrary specific anchor sequence as described previously (*9*). cDNA was RNase H-treated before random amplification by PCR. The resulting products were purified by using MinElute (QIAGEN, Hilden, Germany) and ligated to linkers for sequencing on a GSL FLX Sequencer (454 Life Sciences, Branford, CT, USA). Sequences were clustered and assembled into contiguous fragments (contigs) after trimming of primer sequences, and BLAST analysis was applied to compare contigs (or single reads) at the nucleotide and amino acid levels to the GenBank database (www.ncbi.nlm.nih.gov).

### PCR and Genome Sequencing

Primers were designed that bridged contigs identified by high-throughput sequencing. Additional primers were selected to resequence the genome with special attention to junctions of the P1 and P2 regions. The PCRs were conducted by using HotStar polymerase (QIAGEN) primers at 0.2 µmol/L each and 1 µL of random hexamer-primed cDNA. The draft genome sequence was confirmed by selecting additional primers to generate ≈1 kb products across the entire sequence for direct dideoxy sequencing (Genewiz, South Plainfield, NJ, USA), applying TaKaRa LA Taq polymerase with GC buffer (TaKaRa Bio, Otsu, Japan) to obtain products in areas that proved difficult to amplify because of potential secondary structures or elevated GC content.

### Quantitative TaqMan Real-time PCR

Primers and probes for quantitative real-time PCR were selected within the 5′ untranslated region (UTR) of the turkey hepatitis virus (THV) genome by using Primer Express 1.0 software (Applied Biosystems, Foster City, CA). The primer/probe set THVforward1 5′-CACCCTCTAYGGGCAATGT-3′, THVreverse1 5′-TCAGCCAGTCTATGGCCAGG-3′, and THVprobe1 6FAM-5′-TGGATTCCCATCTCACGCGTCCAC-3′-TMR used in assay 1 (Table 1) was chosen on the basis of the initial THV strains sequenced. Primer THVforward2 5′-CACCCTYYAYGGGCAAATGT-3′ and probe THVprobe2 6FAM-5′-ATTCCCATCTCACGCGTCCAC-3′-TMR were later selected to address sequence variation of additional strains and used with THVreverse1 primer in assay 2 (Table 2). A calibration standard for both assays was generated from strain 2993A by cloning a 571-nt genomic fragment into the pGEM-T Easy vector (Promega Corp., Madison, WI, USA). PCRs were pursued in triplicate by using a StepOnePlus Real-time PCR system (Applied Biosystems), and a standard cycling profile of 45 cycles in a volume of 25 µL containing random hexamer-primed cDNA, 300 nmol/L primer (each), and 200 nmol/L probe. Results were expressed as mean copy number per 300 ng total RNA.

**Table 1:** Real-time PCR measurement of viral sequences in organ samples from turkey poults with turkey viral hepatitis compared with controls, California, USA, 2008–2010*

Poult no.	Age, d	Organ	C_t_	Virus copies†
Infected				
2993A	25	Liver	17.12	5.66 × 10^7^
2993B	25	Liver	17.02	6.22 × 10^7^
2993C	25	Liver	26.98	9.0 × 10^3^
2993D	25	Liver	17.93	2.8 × 10^7^
0091.1	28	Liver	17.24	5.1 × 10
0091.2	28	Liver	17.69	3.43 × 10^7^
0091.3	28	Liver	23.26	2.57 × 10^5^
1813.1	26	Liver	23.48	2.12 × 10^5^
1813.2	26	Liver	21.23	1.53 × 10^6^
1813.3	26	Intestine	20.94	1.97 × 10^6^
0690	30	Liver	34.92	8.98 × 10°
1999	29	Liver	28.44	8.42 × 10^3^
		Pancreas	25.97	7.32 × 10^4^
		Intestine	24.85	1.96 × 10^5^
Control				
1621.1	42	Liver	>36‡	Negative
1621.2	42	Liver	>36‡	Negative
1621.3	42	Liver	>36‡	Negative

*C_t_, cycle threshold. †In 300 ng total RNA. Copy numbers were calculated on the basis of a standard curve generated from cloned target sequences. ‡C_t_ >36 was rated as negative on the basis of the highest dilution of standard representing 5 copies.

**Table 2:** Real-time PCR measurement of viral sequences in samples from turkey poults with turkey viral hepatitis compared with controls, California, USA, 2008–2010*

Poult no.	Age, d	Sample	C_t_	Virus copies†
Infected				
2641.1	29	Cloacal swab	21.68	2.28 × 10^7^
		Bile	31.44	7.58 × 10^4^
		Serum	43.12	1.2 × 10^2^
2641.2	29	Cloacal swab	>44‡	Neg
		Bile	32.5	4.04 × 10^4^
		Serum	38.04	1.67 × 10^3^
2641.3	29	Cloacal swab	>44‡	Neg
		Bile	28.60	3.84 × 10^5^
		Serum	34.18	1.55 × 10^4^
2641.4	29	Cloacal swab	30.6	1.21 × 10^5^
		Bile	27.72	6.48 × 10^5^
		Serum	41.06	3.06 × 10^2^
2641.5	29	Cloacal swab	28.11	5.09 × 10^5^
		Bile	32.8	5.84 × 10^4^
		Serum	41.33	2.71 × 10^2^
394.1	28	Cloacal swab	>44‡	Neg
		Serum	43.27	8.17 × 10^1^
394.2	28	Cloacal swab	40.7	4.55 × 10^2^
		Serum	>44‡	Neg
394.3	28	Cloacal swab	>44‡	Neg
		Serum	>44‡	Neg
394.4	28	Cloacal swab	29.62	2.14 × 10^5^
		Serum	>44‡	Neg
394.5	28	Serum	>44‡	Neg
394.6	28	Serum	>44‡	Neg
394.7	28	Serum	>44‡	Neg
394.8	28	Serum	>44‡	Neg
394. 9	28	Serum	31.07	9.34 × 10^4^
3302.1	39	Serum	36.38	3.08 × 10^3^
3302.2	39	Serum	>44‡	Neg
3302.3	39	Serum	>44‡	Neg
3302.5	39	Serum	>44‡	Neg
Control				
2491.1	32	Cloacal swab	>44‡	Neg
2491.2	32	Cloacal swab	25.67	2.2 × 10^6^
2491.3	32	Cloacal swab	>44‡	Neg
2491.4	32	Cloacal swab	>44‡	Neg
2491.5	32	Cloacal swab	>44‡	Neg
2491.6	32	Cloacal swab	34.74	1.11 × 10^4^
407.1	39	Cloacal swab	>44‡	Neg
407.2	39	Cloacal swab	>44‡	Neg
407.3	39	Cloacal swab	>44‡	Neg
407.4	39	Cloacal swab	>44‡	Neg

*C_t_, cycle threshold; neg, negative. †In 300 ng total RNA. Copy numbers were calculated on the basis of a standard curve generated from cloned target sequences. ‡C_t_ >44 was rated as negative on the basis of the highest dilution of standard representing 5 copies.

### Phylogenetic Analysis

Phylogenetic analyses were performed based on THV P1, 2C/3C/3D, and full polyprotein sequence excluding divergent aa 799–1199. Sequences were aligned to selected members of the *Picornaviridae* family by ClustalW and trees were constructed by using the Molecular Evolutionary Genetics Analysis (MEGA) software version 4.0.2 (*10*). A Jukes-Cantor model was applied to calculate distance, and statistical significance was assessed by bootstrap resampling of 1,000 pseudoreplicate datasets.

### In Situ Hybridization

Viral probes and β-actin control probes were designed and applied according to the QuantiGene ViewRNA protocol by using branched DNA technology (Panomics, Fremont, CA, USA). Twenty viral probes, 17–28 nt long, were selected that cover 500 nt of target sequence in 2B/2C. Forty β-actin probes, 17–26 nt long, were selected covering 873 nt of mRNA sequence. Five-micrometer–thick paraffin-embedded tissue sections were fixed, permeabilized with protease, and hybridized with oligonucleotides conjugated to alkaline phosphatase. After incubation with FastRed substrate, the slides were counterstained with hematoxylin and mounted with coverslips by using Permount (Fisher Scientific, Pittsburgh, PA, USA). Images were acquired by using a Zeiss AX10 Scope AI, ProgRes digital microscope camera and Mac Capture Pro 2.6.0 software (Jenoptik, Jena, Germany).

### Immunohistochemical Analysis

Glass slides with embedded tissues were heated at 56°C for 10 min and washed in citrus clearing agent (Fisher Scientific) to remove paraffin. The sections were rehydrated through graded alcohol solutions. Endogenous peroxidase activity was blocked by incubation in 0.3% H_2_O_2_ diluted in methanol for 30 min. Nonspecific binding was blocked by incubating sections in 10% goat serum in phosphate-buffered saline (PBS) for 1 h at 37°C. Serum specimens from poults with or without disease were added to the sections at 1:1,000 dilutions in PBS for overnight incubation at 4°C. After being washed in PBS, horseradish peroxidase–labeled goat anti-turkey immunoglobulin G (KPL, Gaithersburg, MD, USA) was added at a 1:250 dilution in PBS for 1 h at 37°C. After PBS washes, the Vectastain Elite ABC kit (Vector Laboratories, Burlingame, CA, USA) was used, with 3, 3′-diaminobenzidine tetrahydrochloride as substrate (ImmPACT DAB kit; Vector Laboratories). The sections were counterstained with hematoxylin and dehydrated through graded alcohols. Finally, the slides were mounted with coverslips by using Permount (Fisher Scientific) and examined under a Zeiss AX10 Scope AI light microscope at ×40 magnification (Jenoptik).

## Results

### Identification of THV

**Figure 1:**
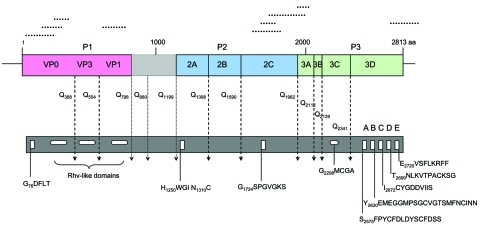
Predicted turkey hepatitis virus (THV) genome organization based on sequence comparison to known picornaviruses**.** Dotted lines above the genome depict the location of the original sequences obtained by high-throughput sequence analysis. Conserved picornaviral motifs and predicted potential cleavage sites along the coding region are indicated in the bar below.

Unbiased high-throughput pyrosequencing of RNA extracted from livers of 4 poults with TVH (animals 2993A, 2993B, 2993C, and 2993D; Table 1) yielded ≈63,100 sequence reads with a mean length of 285 nt. Seven contigs (average length of 674 nt, comprising 105 sequence reads) and 2 singletons (read lengths of 486 nt and 498 nt) that together yielded 3,182 nt of sequence were identified after primer trimming and assembly. Analysis at the nucleotide level was not informative; however, BLASTx analysis revealed significant similarity to picornavirus sequences at the amino acid level. The remainder of the genome was determined from RNA of poult 2993D by RT-PCR with primers linking the individual contigs and singletons (Figure 1). Applying additional primers in various genome regions, a second 9-kb genomic sequence was generated from another diseased poult (animal 0091.1). Sequence analysis showed that the second strain had the same genome organization as strain 2993D and 96.8% aa and 89.9% nt sequence identity (GenBank accession nos. HM751199 and HQ189775).

### THV Genome Organization

The THV genome, comprising >9,040 nt and 2,813 aa, is larger than that of equine rhinitis B virus (genus *Erbovirus*), the largest known picornavirus genome (*11*). The length chiefly reflects the presence of a 1.2-kb sequence at the junction of the P1 and P2 regions in an otherwise typical picornavirus genome (Figure 1). The incomplete 461-nt 5′ UTR includes a 30-nt motif (nt 270–300 of THV) that is identical to duck hepatitis A virus 5′ UTR (*Avihepatovirus* genus), which has a type IV internal ribosome entry site. However, Mfold-modeling of the available sequence did not allow identification of the internal ribosome entry site type present in THV. At the 3′ end, 140 nt of the UTR were recovered (without reaching a poly-A tail) that showed no sequence similarity to other picornavirus 3′ UTRs.

With the exception of a highly conserved Gxxx[T/S] motif (*12*) that in some picornaviruses permits myristoylation, little sequence conservation occurs in the VP0 of THV with respect to other picornaviruses. Cleavage at an upstream Q_73_/A would not generate an N terminal G, cleavage at this site is not supported by NetPicoRNA prediction, and the presence of a leader sequence is unclear (Figure 1; www.cbs.dtu.dk/services/NetPicoRNA). Therefore, as in parechoviruses, hepatitis A virus, and avian encephalomyelitis virus, this site is probably not functional for myristoylation in THV (*13**–**15*). NetPicoRNA analysis did not indicate a VP2/4 maturation cleavage as found in avihepatoviruses, kobuviruses, and parechoviruses. Sequence comparisons of the P1 region, mainly driven by recognizable sequence conservation in VP3 and a few regions in VP1, indicate the highest amino acid identity (20%) with turdiviruses, unclassified picornaviruses recently identified from wild birds (*16*). Homology in P1 to pfam sequence cluster cd00205 “picornavirus capsid protein domain like” (GenBank accession no. PF00073) was observed between amino acid residues 109–267, 391–539, and 622–768 (Figure 1). Potential cleavage sites within P1 are predicted after Q_388_ (VP0/3) and Q_554_ (VP3/1).

Protein 2A motifs in THV are conserved with respect to kobuviruses after a predicted cleavage site Q_1199_. However, the sequence lacks the trypsin-like protease motifs that allow autocatalytic cleavage at the N-termini of enteroviruses and sapeloviruses, as well as the NPGP motif that facilitates C-terminal cleavage in aphthoviruses, avihepatoviruses, cardioviruses, erboviruses, senecaviruses, and teschoviruses. The predicted 2A sequence of THV resembles Hbox-NC motifs in hepatoviruses, kobuviruses, parechoviruses, and tremoviruses (H_1250_WGI, N_1310_C followed by a hydrophobic region L_1332_-V_1350_). The THV 2A protein may be generated by cleavage at conserved protease sites; however, multiple cleavage sites between P1 and P2 are predicted by NetPicoRNA (Figure 1). Cleavage after Q_798_ and Q_1199_ appears likely because it would generate VP1 and 2A products that align with other picornavirus proteins. As a result, 1 or 2 additional proteins (depending on cleavage at Q_880_, Figure 1) may be produced from this genome region that have no homology to any viral product recorded in GenBank. Multiple 2A1, 2A2, or 2A3 protein products with undefined function are described in Ljungan virus, seal picornavirus, and duck hepatitis virus genomes (*17**–**19*). Although we predict, based on alignment analyses, that the C-terminal cleavage of 2A occurs at Q_1398_, this prediction is poorly supported by NetPicoRNA. 2B and 2C have sequence homology to kobuvirus sequences, particularly in a conserved 2C helicase domain G_1724_SPGVGKS that aligns to the PF00910 RNA helicase domain.

Although no sequence homology to any picornavirus record in GenBank was found for THV 3A, 3B displays a conserved tyrosine in position 3 as well as a conserved glycine in position 5. The THV 3C protease contains the active site motif G_2298_MCGA, which is consistent with 3C proteases of other picornaviruses, and shows highest homology to cosaviral 3C (PF00548 3C cysteine protease [picornain] aligning to aa 2153–2321, Figure 1). The identity of a 472-aa sequence at the 5′ end of the genome as THV 3D is supported by homology of aa 2355–2809 to PF00680 RNA-dependent RNA polymerases, and the conservation of positive-strand viral RNA-dependent RNA polymerase motifs A–E (*20*) (Figure 1).

The assembled THV genome sequences were used to reanalyze the initial read library generated by unbiased high-throughput pyrosequencing. This analysis confirmed the presence, and overlap with, the adjacent sequences of divergent 2A and 3A region reads in the initial dataset.

**Figure 2:**
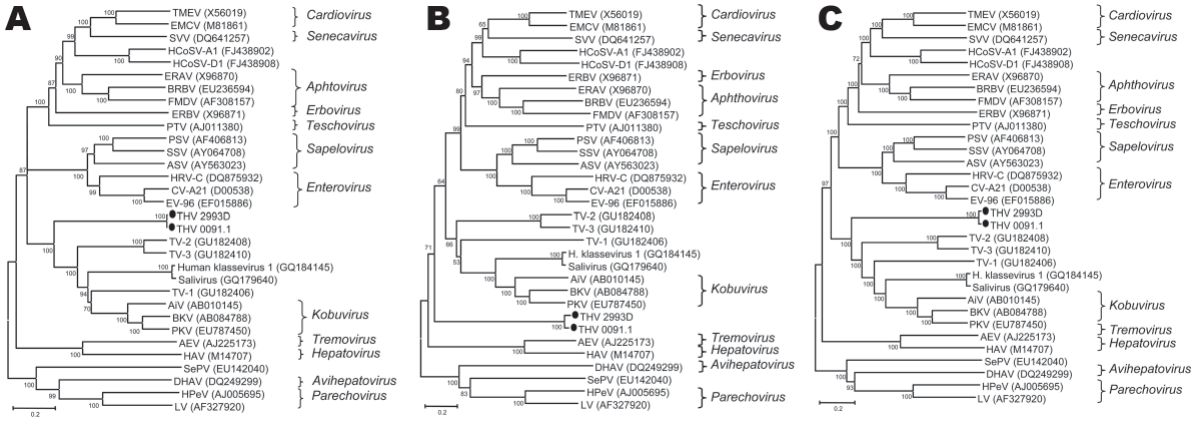
Relationships between turkey hepatitis virus (THV) and other picornaviruses. The phylogenetic analyses were based on amino acid sequences of the combined 2C, 3C, and 3D regions (A), the P1 region (B), and complete coding regions, excluding divergent aa 799–1199 (C). Representative sequences from different picornavirus genera and recently discovered, unclassified viruses were obtained from GenBank; accession numbers are indicated. Bootstrap values are given at the respective nodes; scale bars indicate number of amino acid substitutions per site. TMEV, Theiler’s murine encephalomyelitis virus; EMCV, encephalomyocarditis virus; SVV, Seneca Valley virus; HCoSV-A1, human cosavirus A1; HCoSV-D1, human cosavirus D1; ERBV, equine rhinitis B virus; ERAV, equine rhinitis A virus; BRBV, bovine rhinitis B virus; FMDV, foot-and-mouth disease virus; PTV, porcine teschovirus; PSV, porcine sapelovirus; SSV, simian sapelovirus; ASV, avian sapelovirus; HRV-C, human rhinovirus C; CV-A21, coxsackievirus A21; EV-96, enterovirus 96; TV-2, turdivirus 2; TV-3, turdivirus 3; TV-1, turdivirus 1; AiV, Aichi virus; BKV, bovine kobuvirus; PKV, porcine kobuvirus; AEV, avian encephalomyelitis virus; HAV, hepatitis A virus; SePV, seal picornavirus; DHAV, duck hepatitis A virus; HPeV, human parechovirus; LV, Ljungan virus.

Phylogenetic analyses based on amino acid sequence of the most informative genome regions 2C/3C/3D, the P1 region, and the full polyprotein sequence (excluding the nonconserved aa 799–1199) showed THV as a distinct species separate from classified genera (Figure 2). The 2C/3C/3D analysis indicates THV in an ancestral position to kobuviruses, klasseviruses, and turdiviruses, the viruses most related to THV.

### THV RNA Load in Liver, Bile, Serum, and Cloacal Swab Specimens

Two TaqMan real-time PCR assays, both targeting the 5′ UTR, were developed to quantitate viral RNA load in affected animals. Assay 1 (Table 1) was replaced by assay 2 (Table 2) after the characterization of additional THV strains indicated divergent sequences.

In the liver samples from turkeys with TVH, viral RNA typically exceeded 10^5^ copies/300 ng of total RNA; only 1 animal had a lower load (animal 0690, Table 1). No viral RNA was detected in livers from non-diseased control animals. Analysis of animals 1813.3 and 1999 indicated presence of the virus in the intestine and pancreas as well as in the liver. Cloacal swab samples, bile, and serum from 28-, 29- and 39-day-old poults with disease were also analyzed. Five of 9 cloacal swab specimens from TVH-affected animals were positive for viral RNA (Table 2). Viral RNA >10^4^ copies/300 ng total RNA was detected in the bile of all 5 poults with TVH tested. Viral RNA was also found in serum samples from 8 of 18 poults with TVH.

Cloacal swabs were analyzed from 32- and 39-day-old turkey poults that did not have TVH according to histopathologic findings. Viral RNA was detected in 2 of 10 animals tested (Table 2).

### Localization of THV RNA and Protein in Liver

**Figure 3:**
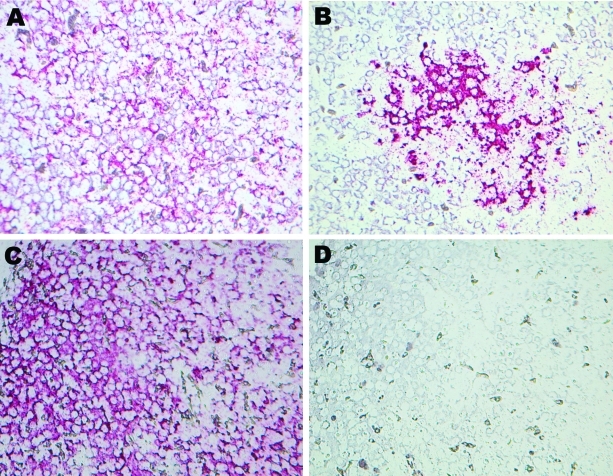
Results of in situ hybridization experiments. Hybridization of β-actin–specific and turkey hepatitis virus (THV)–specific oglionucleotide probes with FastRed staining on hepatitis-affected liver tissue from poult 2993A (A and B, respectively) and on nondiseased liver tissue from poult 1927B (C and D, respectively). Brightfield microscopy images; original magnification ×40.

The distribution of THV RNA was examined in liver sections of affected and unaffected poults by in situ hybridization. THV signal was found in the cytoplasm of hepatocytes of affected poults. No signal was observed in healthy poults (Figure 3). A hybridization signal with a control β-actin probe was present in both affected and healthy poults; however, β-actin signal was less pronounced in affected poults. In situ hybridization also indicated viral RNA in 1 intestinal sample from the 0091 animals (not shown), in line with the real-time PCR data obtained for animals 1813.3 and 1999 (Table 1).

**Figure 4:**
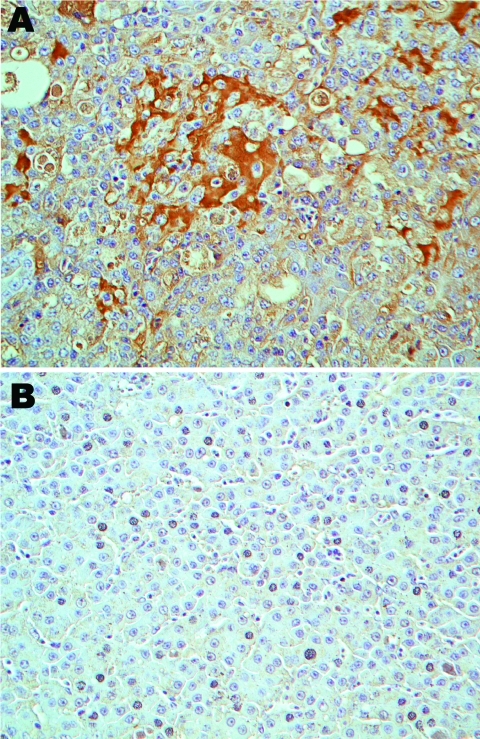
Immunohistologic staining of liver tissues with serum from a turkey poult with turkey viral hepatitis (TVH). Serum sample from PCR-positive poult 394.9 demonstrates turkey hepatitis virus (THV) antigens in clusters of cells in liver tissue of TVH-affected poult 2993A (A) but not in liver sections from nondiseased poult 1927B (B). Sections were counterstained with hematoxylin. Brightfield microscopy images; original magnification ×40.

Serum from TVH-affected poult 394.9, which was positive by PCR for THV RNA (Table 2), was used on paraffin-embedded tissues for immunohistochemical analysis. The serum showed reactivity when tested in livers from affected poults. No reactivity was observed with healthy poults (Figure 4).

## Discussion

The *Picornaviridae*, a family of small, nonenveloped viruses with a positive-sense, single-strand RNA genome, currently consist of 12 genera. Recent additions to the family include salivirus NG-J1 and human klasseviruses identified in pediatric stool samples (*21**–**23*), cosaviruses (*24**,**25*), and an unclassified seal picornavirus 1 (*17*). Our phylogenetic analyses indicate that THV, although distantly related to viruses of the *Kobuvirus* genus as well as to unclassified klasseviruses/saliviruses and recently identified turdiviruses, is distinct from known picornaviruses.

Most peculiar is the unique 2A region of THV. THV appears to encode multiple 2A products. Although multiple 2A products have also been described for Ljungan virus, seal picornavirus, and duck hepatitis virus (*17**–**19*), the 2A region of THV shows no sequence homology to these products or to any other picornavirus sequence currently in GenBank. Also remarkable is the lack of sequence homology of THV 3A to picorna- or other virus sequences. Whereas VP3 (representing P1) is closest to turdivirus 1 (30% aa identity), 2B/C (representing P2) is closer to Aichi virus (28%) and 3C/D (representing P3) is closer to turdivirus 3 (39%). Thus, THV shows classic features of known picornaviruses but also unique features that do not support inclusion of THV into existing taxa of the *Picornaviridae* family.

Turkey poults with TVH may have diarrhea and pancreatitis as well as hepatitis. Picornavirus particles in the feces of animals with TVH have been described (*26**,**27*). Accordingly, we detected THV RNA in the intestine, pancreas, bile, and cloaca samples as well as the liver. These findings are consistent with a fecal–oral route for transmission. We also detected THV RNA in cloacal swab samples from 2 of 10 asymptomatic poults. Because these animals were housed on a farm with history of TVH, this finding is consistent with reports suggesting subclinical infections (*2**,**6*). The advent of a noninvasive screening test for THV may aid in disease containment.

We molecularly characterized a picornavirus in turkey viral hepatitis and linked it to disease through measurements of load and tissue distribution, viremia and a humoral immune response to the agent. On the basis of the data presented here, we suggest that THV represents a new species in the order Picornavirales and a likely candidate for causing TVH.
